# Medical Genetics – Special issue dedicated to the 35^th^ anniversary of the Medical Genetics Service, Hospital de Clínicas de Porto Alegre, Brazil

**DOI:** 10.1590/1678-4685-GMB-2019-00040001

**Published:** 2019-04-11

**Authors:** Roberto Giugliani

**Affiliations:** 1 Medical Genetics Service, Hospital de Clínicas de Porto Alegre, Brazil; 2 Department of Genetics, Universidade Federal do Rio Grande do Sul, Porto Alegre, RS, Brazil; 3 Programa de Pós-Graduação em Genética e Biologia Molecular, Universidade Federal do Rio Grande do Sul, Porto Alegre, RS, Brazil; 4 National Institute of Population Medical Genetics - INAGEMP, Porto Alegre, RS, Brazil

In January 1982, the Medical Genetics Unit of Hospital de Clínicas de Porto Alegre (HCPA) began its history in Brazil. It was elevated to a Medical Genetics Service (SGM) in 1996, and by the end of 2016, it celebrated its 35^th^ anniversary. This is a tale of continued progress, grounded in the idea that patient care, education, and research grow more and grow better if they are developed in harmony.

In the SGM/HCPA, several units of the Federal University of Rio Grande do Sul state – UFRGS (Institute of Biosciences, Institute of Basic Health Sciences, Faculty of Medicine, Faculty of Pharmacy), and several postgraduate programs (Biochemistry, Genetics, Medical Sciences, Child and Adolescent Health, and Pharmaceutical Sciences) have become intrinsically interconnected. This heterogeneous and plural composition, acting around the central theme “Medical Genetics,” became the brand of the group, which interacts transversally with numerous areas of the HCPA and has several associated sectors in the Experimental Research Center (Laboratory of Genetic Identification, Laboratory of Genomic Medicine, Gene Therapy Center, and Brain Laboratory) and the Clinical Research Center.

Thus, the different academic, technical, and scientific professionals involved in patient care activities, which have always been the central axis of the mission of SGM/HCPA, received the support of undergraduate scientific initiation trainees, MSc and PhD students, and postdoctoral fellows linked to numerous projects of scientific and clinical research that were developed. In this virtuous circle, the different components supported each other and resulted in a very impressive number of consultations, laboratory tests, clinical research protocols, scientific articles, and human resource formation on various levels.

A solid educational platform with several specialized courses was progressively developed with an accredited medical residency program and a portfolio of different training programs, which were highly demanded by professionals from Brazil and abroad, like the Latin American School of Human and Medical Genetics (ELAG). Specialists and investigators from all corners of the world visited SGM/HCPA and contributed with their experience and expertise throughout these 35 years.

The leadership position was also expressed by the presidency of members of the SGM/HCPA in several scientific societies of this field (Brazilian Society of Medical Genetics (SBGM), Latin American Society of Inborn Errors or Metabolism and Neonatal Screening (SLEIMPN), Latin American Network of Human Genetics (RELAGH), and Brazilian Society of Biochemistry and Molecular Biology (SBBq), among others, and by the promotion of memorable national and international events in the area. It was also expressed by hosting the headquarters of a National Institute of Science and Technology (National Institute of Population Medical Genetics - INAGEMP) and the editorial team of an international scientific journal (Journal of Inborn Errors of Metabolism and Screening - JIEMS).

Considered the most complete service of its kind in the Latin American continent, it included the combined development of the main pillars of medical genetics (clinical genetics, cytogenetics, biochemical genetics, and molecular genetics), while integrating it with specific programs (monitoring of birth defects, prenatal diagnosis, oncogenetics, neurogenetics, population medical genetics, and information and diagnostic networks), and with education, training and research activities.

In 2004, the SGM/HCPA was designated by the World Health Organization as a Collaborating Center for the Development of Medical Genetic Services in Latin America, and, in 2016, it was qualified by the Brazilian Ministry of Health as a Reference Service in Rare Diseases.

**Figure 1:**
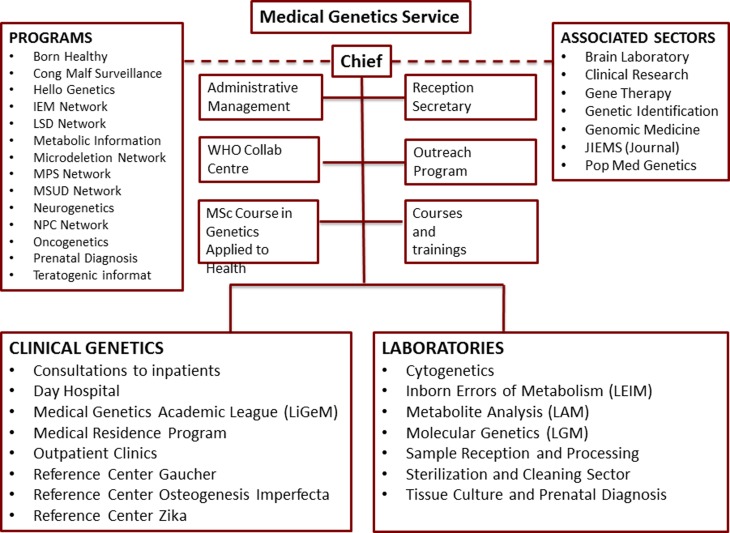
Medical Genetics Service, HCPA – Organizational Chart (December 2016).

There is nothing better to mark the 35^th^ anniversary of this successful history (summarized in Figure 1, which illustrates its structure in December 2016) than a collection of 15 scientific articles that, without proposing to be a systematic representation of its activity, comprehensively illustrates the innumerable contributions of the group to the medical genetics field in Brazil and internationally.

*Guest Editor*

*Roberto Giugliani,*

*Medical Genetics Service, Hospital de Clínicas de Porto Alegre, Brazil.*

*Guest Editorial Assistants*

*Mariluce Riegel and Francyne Kubaski*

*Medical Genetics Service, Hospital de Clínicas de Porto Alegre, Brazil.*

